# Methodological approach for predicting and mapping the phenological adaptation of tropical maize (*Zea mays* L.) using multi-environment trials

**DOI:** 10.1186/s13007-018-0375-7

**Published:** 2018-12-07

**Authors:** Henri E. Z. Tonnang, Dan Makumbi, Peter Craufurd

**Affiliations:** 1 International Institute of Tropical Agriculture (IITA), 08 BP 0932 Abomey Calavi, Cotonou, Benin; 2 International Maize and Wheat Improvement Center (CIMMYT), ICRAF House, United Nations, Avenue, Gigiri, P. O. Box 1041-00621, Nairobi, Kenya

**Keywords:** Temperature dependent model, Environmental variables, Length of growing period, Landscape

## Abstract

**Background:**

The phenological development of the maize crop from emergence through flowering to maturity, usually expressed as a rate (i.e. 1/duration), is largely controlled by temperature in the tropics. Maize plant phenological responses vary between varieties and quantifying these responses can help in predicting the timing and duration of critical periods for crop growth that affect the quality and quantity of seed. We used routine multi-environment trials data of diverse tropical maize varieties to: (1) fit 82 temperature dependent phenology models and select the best model for an individual variety, (2) develop a spatial framework that uses the phenology model to predict at landscape level the length of the vegetative and reproductive phases of diverse varieties of maize in different agro-ecologies. Multi-environment trial data of 22 maize varieties from 16 trials in Kenya, Ethiopia, and Sudan was analyzed and the Levenberg–Marquardt algorithm combined with statistical criteria was applied to determine the best temperature-dependent model.

**Results:**

The Briere model, which is not often used in plant phenology, provided the best fit, with observed and predicted days to flowering showing good agreement. Linking the model with temperature and scaling out through mapping gave the duration from emergence to maturity of different maize varieties in areas where maize could potentially be grown.

**Conclusion:**

The methodology and framework used in the study provides an opportunity to develop tools that enhance farmers’ ability to predict stages of maize development for efficient crop management decisions and assessment of climate change impacts. This methodology could contribute to increase maize production if used to identify varieties with desired maturity for a specific agro-ecology in in the targeted regions.

**Electronic supplementary material:**

The online version of this article (10.1186/s13007-018-0375-7) contains supplementary material, which is available to authorized users.

## Introduction

The method of using temperature and time as factors to describe the development of plants and insects was proposed and has been continuously studied for over 300 years [1]. The concept began with the French naturalist [1] who suggested that temperature variations were probably one of the causes of the changes in plant and insect phenology. They proposed summing up the mean daily air temperature for an identical number of months in a location, which led to the birth of the degree-day concept as the values of the developmental rate of plants obtained from year to year were roughly constant [1]. The degree-day equation was later modified by several authors [2–4]. As the concept of degree-day evolved, a new hypothesis arose stating that the rate of chemical reactions is either doubled or tripled for each 10 °C rise in temperature [5]. This idea was expressed in form of a constant called Q_10_ [5]. Towards the end of the 18th century, the principle started to influence the formulation of plant and insect development rate functions [5].

Mathematical, statistical and machine learning approaches are commonly used to enhance the understanding of plant architecture, growth, development, and interactions with the environment. A single-parameter compartmental model was used to describe the transport of fluoride in living plants [6]; and an ensemble of statistical metrics was applied to quantify large sets of plant transcription factor binding sites [7]. In Qiongyan et al. [8] a neural network was developed to detect the spikes of wheat plants, and an algorithm with deep learning method was applied for counting the leaf of rosette plants [9]. Increasingly, the formulation of mathematical expressions to represent the development of plants and insects continued to progress centered on the postulation that “within certain ranges, as temperature (*Te*) decreases, the rate of development slows and, if the temperature drops low enough, development will cease at the organism’s lower developmental threshold; often called the base temperature (*Tb*). As temperature increases, development rate increases until temperature reaches the optimum temperature (*To*), above which the development rate decreases and eventually ceases at a value called maximum temperature (*Tm*)”. Inspired by this hypothesis, various models have been developed, each with strengths and weaknesses [10]. Largely, these models derived the relationship between the development of plants and insects and temperature either empirically or through process-based methodology. Empirical functions are formulated and parameterized using the same measured phenomenon as the phenomenon to be derived [11]. In contrast process-based functions are mathematical expressions formulated and parameterized using biological knowledge such as the enzyme kinetics to predict the time from planting to anthesis [11].

Climate has a considerable impact on the distribution and abundance of plants and other organisms, and the mathematical depiction of the climatic effect on crop phenology has been of significant interest among scientists [12, 13]. Temperature is the most important climate variable determining plant phenology (via rate of development) and plant distribution (via base and maximum temperature limits to survival) [14, 15]. Understanding the phenology of maize can therefore help to define crop adaption to a region or site (i.e. its ability to mature and set seed within a growing season). In maize, it can also help hybrid seed production by determining appropriate planting dates of lines to ensure flowering synchrony [15, 16]. Therefore, the accurate prediction of phenological development is fundamental to define crop adaption and yield potential [15, 17].

Despite the abundance of literature [15, 18, 19] on the use of temperature dependent models for the prediction of maize developmental phases, few studies have been conducted on tropical maize in sub Saharan Africa (SSA). Furthermore, in order to get the best possible prediction of phenology for individual varieties across space and time, a range of well-known and well-tested temperature-dependent models for plant and insect development should be evaluated and made available through an open-source modeling framework. If this framework can be based on existing and annually or routinely conducted breeding or variety-testing trials, then the process can become institutionalized. Models can then be used to predict the adaptation of different maize varieties spatially and temporally across major maize growing agro-ecologies, information that is important in efforts to improve maize yields. The objectives of this study were therefore to: (1) to evaluate 82 different temperature dependent models to predict the phenological development of 22 diverse tropical maize varieties using existing multi-environment trial data; and (2) develop a spatial framework that uses the phenology model to predict the period from sowing to flowering or maturity across sites and agro-ecologies, i.e. to map the adaptation of different varieties.

## Materials and methods

### Maize varieties and test locations

**Fig. 1:**
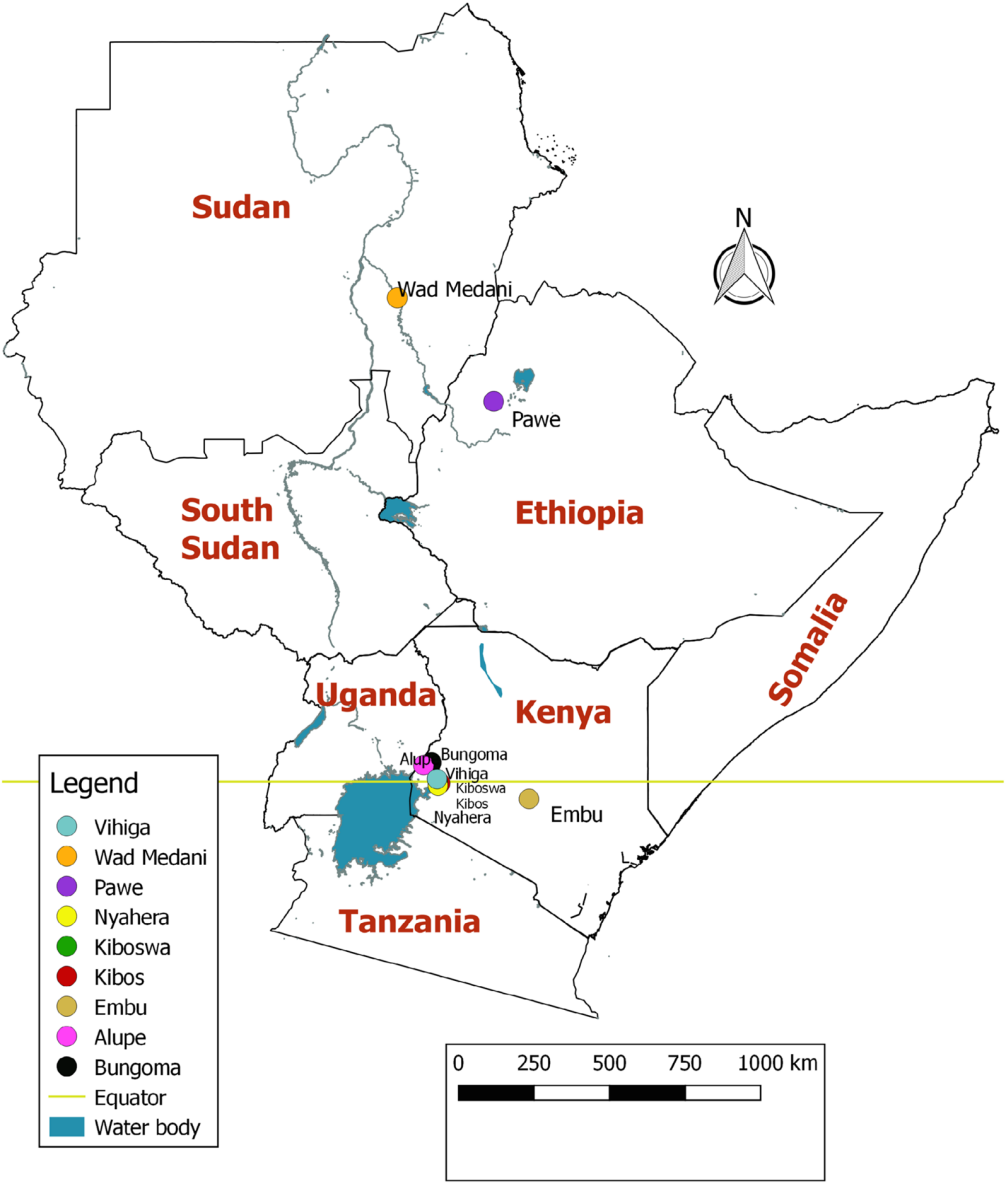
Locations of early and intermediate open-pollinated maize varieties trials conducted in Kenya, Ethiopia and Sudan

Twenty-two (22) early and intermediate maturity open-pollinated maize varieties were planted in nine different locations in Kenya, Ethiopia, and Sudan in 2004 (a total of 16 trials) as described in Table 1 and Fig. 1 (adapted from [16]). In each of these trials the date when 50% of the plants in a plot: (1) emerged; (2) had a tassel (male); (3) had a silk (female) and (4) were physiologically mature (black layer formation) was recorded. Selection of experimental field data for model development and calibration was conducted using altitudinal change (400–1600 m) to represent a wide range of temperature values (11–20 °C minimum; 24–37 °C maximum). Six sites covering the maximum range of temperature were selected for model development and calibration, namely Alupe, Embu, Kibos and Bungoma, Pawe, Wad Madani. The remaining three sites, namely Kiboswa, Nyahera and Vihiga, were used for independent model evaluation. In model development about 1/3 of data are typically used for independent validation.

**Table 1:** Description of the locations where data used to develop, calibrate and validate the models for the selected maize varieties was collected as described in [16]

Locations (latitude–longitude)	Altitude (m)	Country	Soil characteristics	Rainfall during cropping season (mm)	Temperature (°C)		Data use
					Minimum	Maximum	
Vihiga (0°32′S–34°47′E)	1629	Kenya	Friable clay	550	14.1	27.0	Model evaluation
Nyahera (0°1′S–34°44′E)	1548	Kenya	Friable clay loam	520	17.0	29.4	Model evaluation
Kiboswa (0°1′S–34°44′E)	1532	Kenya	Friable clay loam	520	17.0	29.4	Model evaluation
Embu (0°30′S–37°27′E)	1504	Kenya	Clay loam	346	13.9	24.6	Model development and calibration
Bungoma (0°33′S–34°33′E)	1374	Kenya	Clay loam	374	11.4	25.4	Model development and calibration
Kibos (0°2′S–34°48′E)	1193	Kenya	Sandy loam	547	16.3	30.7	Model development and calibration
Alupe (0°30′N–34°7′E)	1153	Kenya	Sandy clay loam	679	15.8	28.6	Model development and calibration
Pawe (11°09′N–36°09′E)	1100	Ethiopia	Clay loam	878	16.6	33.4	Model development and calibration
Wad Medani (14°23′N–33°31′E)	400	Sudan	Clay	119	20.3	36.9	Model development and calibration

### Temperature datasets and shape file of suitable soil and weather conditions

Two types of temperature data were used, daily temperatures for model development and monthly gridded temperature for mapping. Daily minimum and maximum temperature were obtained from weather stations in the vicinity of field trials [16]. Maize phenology data were collected during two growing seasons; March to August 2004 characterized by higher rainfall, and September 2004 to January 2005 characterised by lower rainfall. The data were used to develop the phenology model. Gridded average monthly minimum and maximum temperature for Kenya were obtained from WorldClim (http://www.worldclim.org/) and the Climate Change, Agriculture and Food Security (CCAFS) (http://www.ccafs-climate.org) databases. The data are organized in layers (grids) with a spatial resolution of 30 s that is equal to 0.9 × 0.9 km.

The shape file of suitable soil and weather conditions for maize production used in this study was obtained from HarvestChoice [20]. It is a spatially disaggregated production statistics database of derived from the Spatial Production Allocation Model (SPAM). The resolution is 5 arc-minute grid cells [20].

### Assumptions

1. Temperature and time were considered as the primary factors affecting the rate of maize development. Photoperiod or daylength can also affect development (maize is a quantitative short-day plant), but breeders select against this trait and most tropical maize varieties are effectively insensitive to photoperiod. Furthermore, Kenya straddles the equator and the variation in photoperiod is very small [21, 22].
2. It was assumed that the trials were grown with adequate nutrients and water and did not suffer adverse stress based on the yields obtained [16]. In general phenological development is only adversely affected by very severe stress [16].
3. Maize plant developmental cycle was divided into two phases: the vegetative phase (VP), starting from emergence to tasseling/silking (flowering), and the reproductive phase (RP), which is from flowering to physiological maturity (PM). It was assumed that the duration of the VP and RP were the same in any given variety based on previous experience. The overall developmental rate per phase for the whole crop was estimated by accumulating the daily development rate values:
  1 $$D = \sum\limits_{0}^{t} {r(T(t),P)\Delta t}$$
  where development *D* is a function of temperature *T* which in turn is a function of time *t*, *r* is the development rate, Δ*t* is the time increment and *P* the vector of parameter values [23]. The time step for the model was fixed to a day.

4. Phenology was predicted spatially only in areas where maize is known to be grown, based on suitability maps and known maize areas.

### Models

According to [24] development is the process of cellular differentiation manifested by different life-phases of an organism. Development time is the duration between life-phases and growth is the increase in biomass resulting from the development. Selecting a mathematical expression for representing the phase of development of a crop is challenging [25]. This study applied 82 temperature-dependent nonlinear equations (Additional file 1: Table S1) which have been used in agricultural production, either in the context of insect phenology modeling or crop development [25, 26].

### Estimation of the model parameters

An important step in conducting this study was to evaluate how well the selected models (out of the 82 tested) fit the observed data. We used the Levenberg–Marquardt (LM) algorithm [27] because it combines both the ‘steepest descent’ and the Gauss–Newton method to evaluate the models. This algorithm iteratively locates the minimum of a function that is expressed as the sum of squares of the nonlinear model [27]. After initialization of the models, the LM algorithm was launched and a goodness of fit procedure was used to find the parameters of the model. The model parameters were estimated by fitting equations to the recorded data from multi-environment trials. A program written in R computing language [28] provided an interactive processing, in which initial values of the model parameter are entered; and once the LM algorithm is launched, the values of the parameters are optimized based on the matching of model output to observed data following the ‘steepest descent’ and the Gauss–Newton methods. Overall, the process is similar to the functioning of LEAF-E; a tool developed to analyze grass leaf growth using function fitting [29].

### Goodness of fit and selection criteria of the model

No single method exists to best assess the goodness of fit of an individual model to specific data. Therefore visual assessment and the coefficient of determination *R*^2^*_Adj* [25, 30] were used. The *R*^2^*_Adj* is estimated as follows:

2 $$R^{2} \_Adj = 1 - \left( {1 - R_{z}^{2} } \right)\left( {\frac{n - 1}{n - k - 1}} \right)$$

where, *n* is the number of observations, *k* is the number of parameters of the *z*th sub-model, and $R_{z}^{2}$ is the correlation coefficient obtained by the following expression:

3 $$R_{z}^{2} = 1 - \sum\limits_{j = 1}^{n} {\left( {y_{j} - \hat{y}_{jz} } \right)^{2} - } \sum\limits_{j = 1}^{n} {\left( {y_{j} - \bar{y}} \right)^{2} }$$

where, $\bar{y}$ is the observed median, y the observed mean and $\hat{y}_{ji}$ is the *j*th predicted value from the *i*th function.

The best-fitted model is selected by examining the residuals and comparing Akaike’s Information Criterion (*AIC*) and the Model Selection Criterion (*MSC*) [25]. The mathematical expressions of the two criteria are:

4 $$AIC = n\ln - \left( {\sum\limits_{i = 1}^{n} {w_{i} \left( {Y_{{obs_{i} }} - Y_{{est_{i} }} } \right)^{2} } } \right) + 2p$$

5 $$MSC = \ln \left( {\frac{{\sum\nolimits_{i = 1}^{n} {w_{i} \left( {Y_{{obs_{i} }} - \bar{Y}_{est} } \right)^{2} } }}{{\sum\nolimits_{i = 1}^{n} {w_{i} \left( {Y_{{obs_{i} }} - \bar{Y}_{{est_{i} }} } \right)^{2} } }}} \right) - \frac{2p}{n}$$

where, *n* is the number of observations, $Y_{{obs_{i} }}$ and $Y_{{est_{i} }}$ are the observed and estimated values for the *i*th observation, *p* is the number of parameters, and *w*_*i*_ is the weight required for each observation. In the case of nested models, *F* test was used to check whether the addition of parameters has a statistically significant contribution to the model before selection [25].

### Best-fit model: Briere et al. [31]

Although all the 82 models were fitted with the available multi-environment trial datasets of different maize varieties, herein we report only two models, which provided the best fit to the selected maize varieties based on criteria outlined above. The full set of models is available in a in the Additional file 1: Table S1.

The Briere model was developed based on four principles: (1) the option to estimate the lower and upper temperature thresholds; (2) the inclusion of an asymmetry in relation to the optimum value of temperature where the developmental rate is highest; (3) the presence of an inflection point; and (4) the introduction of a decay in development rate at temperatures above the optimum temperature [31]. With these considerations, the lower and the upper thermal limits were unequivocally integrated in the equation to represent two important parameters of the model, *T*_*b*_ and *T*_*m*_, respectively [31]. To get decay at high temperatures, a square root was included to allow a high slope when the values of temperatures approach *T*_*m*_. By combining the products of different powers of temperature, an inflection point occurs yielding the Briere_1 model with the following mathematical expression.

6 $$r(T) = a*T\left( {T - T_{o} } \right)\left( {\sqrt {T_{max} - T} } \right)$$

A second model, Briere_2, was derived from Briere_1 model by replacing the square root with a general power equal to *d *= *1/µ*, where *µ* is the new parameter and *a* is an empirical constant [31]. The Briere_2 equation is as follows:

7 $$r(T) = a*T\left( {T - T_{o} } \right)\left( {\sqrt {T_{max} - T} } \right)^{d}$$

### Evaluation of the model

The evaluation of the model in this study was defined as the level to which the selected model correctly predicted the number of days a maize variety takes from emergence to flowering and from flowering to physiological maturity at different, independent locations. To carry out this procedure, independent data recorded at three typical sites in Kenya (Kiboswa, Nyahera and Vihiga) were used. Flowering and maturity dates were predicted for a standard and common sowing date for each variety from the models developed using data from the other six sites and observed and predicted values compared.

### Spatial predictions of the duration of maize phenology

A Kenya boundaries shape file was divided into grids or cells. To predict the number of days each phase of maize development takes to be completed in an individual cell, the temperature-dependent mathematical expression obtained during the modeling step was run at each individual cell of Kenya using a typical sowing date. The gridded temperature datasets were loaded into the computer, simultaneously extracted from the database and then organized in matrix format using longitude as column and latitude as a row. A point object picks the temperature-dependent mathematical expression of the maize vegetative and reproductive phases, and these are consecutively applied in each geographical coordinate of the grid. After replacing the values of temperature in the phenology model in each grid, a new matrix with the values of the development rate in the respective geographical coordinates was computed. These values are then inversed (1/development rate) to estimate the number of days used to complete each phase of maize development. The results are converted into ASCII files and transferred to an open source software Q-GIS [32] for visualization.

## Results

The multi-environment datasets were used to fit a total of 82 temperature driven models (Additional file 1: Table S1). However, only the model of Briere et al. [31] was selected to calculate the duration of vegetative and reproductive phases of 22 different maize varieties in Kenya. In addition to describing the data accurately (*R*^2^*_Adj* and *R*^2^ > 0.82 for all tested varieties), the Briere model was able to provide biologically meaningful estimates of *T*_*b*_ and *T*_*m*_ for development of 8–10 °C, and 38–40 °C, respectively.

**Fig. 2:**
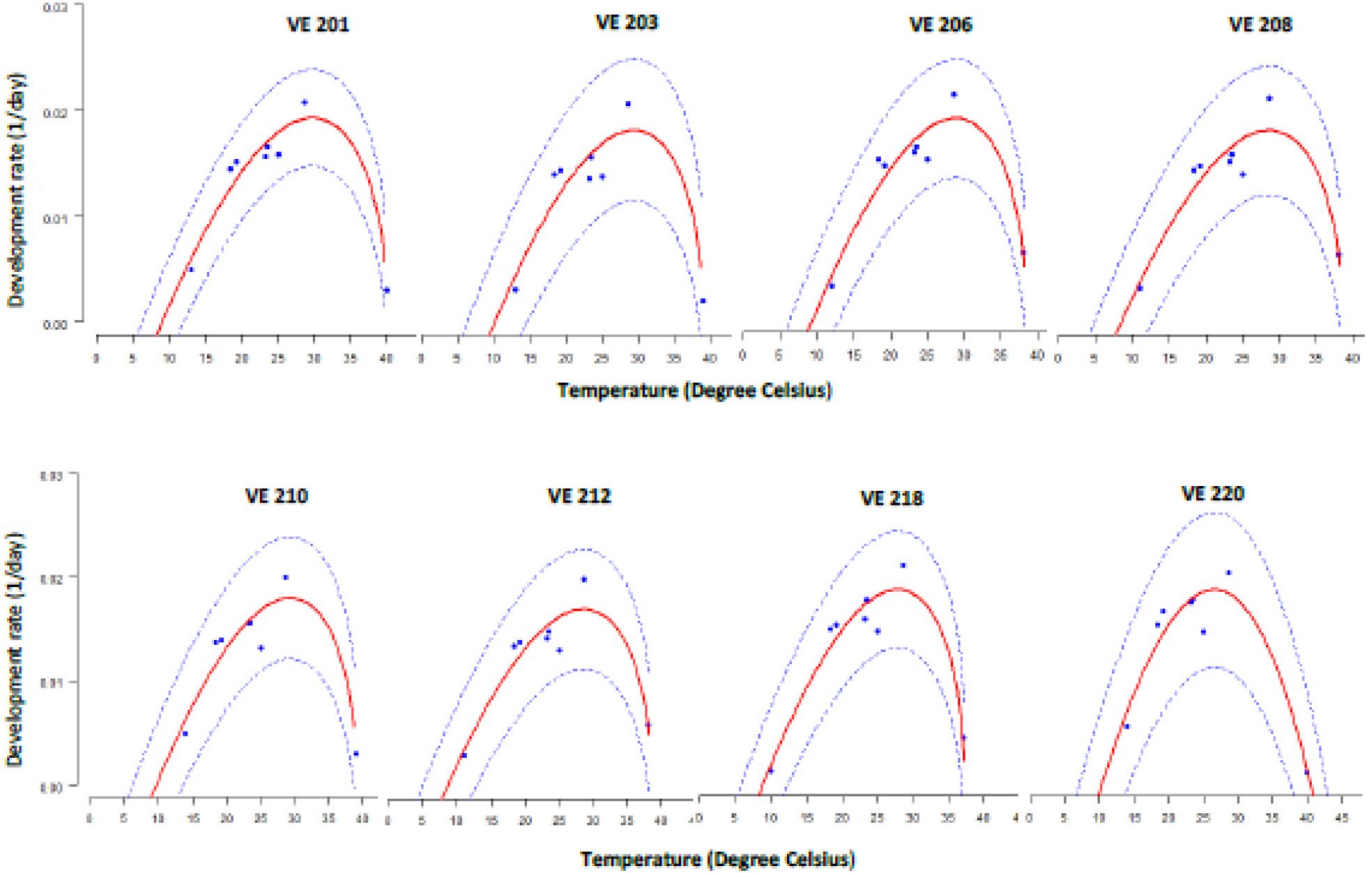
Temperature dependent models of development rate (1/development time) for maize varieties VE 201, VE 203, VE 206, VE 208, VE 210, VE 212, VE218, and VE 220 respectively

Parameter estimates from the Briere_1 and Briere_2 models of eight-selected maize varieties are presented in Table 2. The estimated *T*_*b*_ ranged from 8.1 to 10.1 °C while *T*_*m*_ ranged from 34.3 to 40.4 °C. The adjusted R^2^ was high for all varieties (0.83–0.91). The root mean square error (RMSE) for each variety’s development rate ranged from 0.0014 to 0.0022, which further indicated the ability of the model to predict the duration of individual maize variety’s phases of phenology. Although the selected model did not explicitly estimate the optimum temperature at which the developmental rate is highest, graphical visualization (Fig. 2) showed that these values were between 23 and 32 °C, consistent with the literature, depending on the variety.

**Table 2:** Estimated parameters of Briere—1 and Briere—2 models [23] for eight maize varieties covering the vegetative and reproductive phases of the crop development

Variety	*T*_*b*_ (°C)	*T*_*m*_ (°C)	*a*	AIC	R^2^	MSC	RMSE
VE 201	8.9826 (0.0011)^a^	40.1123 (0.6633)	0.0003 (0.0001)	− 74.3393	0.9389	− 0.8117	0.0014
VE 203	10.1398 (0.0008)	39.0562 (0.1416)	0.0003 (0.00002)	− 68.7885	0.8794	− 0.8737	0.0020
VE 206	9.515 (0.1002)	38.4895 (0.1000)	0.0003 (0.00002)	− 71.7878	0.9010	− 0.8500	0.0017
VE 208	8.5664 (0.1008)	38.587 (0.2510)	0.0003 (0.0001)	− 70.7438	0.8763	− 0.8719	0.0018
VE 210	9.6988 (0.0310)	39.1233 (0.0523)	0.0003 (0.00001)	− 70.3806	0.8848	− 0.8690	0.0018
VE 212	9.0336 (0.0608)	37.2379 (0.1154)	0.0003 (0.00002)	− 72.3643	0.9242	− 0.8238	0.0016
VE 218	8.1142 (0.1004)	40.0585 (0.0001)	0.0029 (0.00004)	− 70.9964	0.9020	− 0.8597	0.0017

Variety	*µ*	*T*_*b*_ (°C)	*T*_*m*_ (°C)	*a*	AIC	R^2^	MSC	RMSE
VE 220	1.0307 (0.000001)	10.0339 (0.000001)	40.4344 (0.0001)	0.00004 (0.000001)	− 65.2251	0.8765	− 1.1314	0.0022

^a^Numbers in parentheses are standard errors

The number of days taken from emergence to flowering, and from flowering to physiological maturity, for the 22 maize varieties at three independent data sets (locations) used for model evaluation is presented in Table 3. The range in observed flowering durations was between 48 and 80 across sites and varieties. Comparison of the duration obtained from model outputs with field trial results showed good agreement (*r* = 0 .892**, Adj R^2^ = 0.786 for Kiboswa; *r* = 0.712**, Adj R^2^ = 0.482 for Nyahera; and *r *= 0.920**, Adj R^2^ = 0.838 for Vihiga) suggesting that the model predicted phenology well and could be used with confidence to predict phenology spatially. The number of days from sowing to emergence is approximately 7–10 days and also varies with temperature. Adding this to the duration of the vegetative and reproductive phases provides gives the length for the growing period of the 22 varieties of maize in any location.

**Table 3:** Observed and predicted number of days taken by each maize variety during the vegetative and reproductive phases of the crop development at three locations used for model evaluation

Varieties names	Locations (latitude–longitude)					
	Kiboswa (0°1′S–34°44′E)		Nyahera (0°1′S–34°44′E)		Vihiga (0°32′S–34°47′E)	
	Observed (days)	Model (days)	Observed (days)	Model (days)	Observed (days)	Model (days)
VE-200	56	52 (03)^a^	73	70 (03)	79	78 (02)
VE-201	56	56 (01)	73	72 (03)	76	75 (02)
VE-202	61	59 (03)	73	71 (02)	78	78 (01)
VE-203	66	62 (02)	79	75 (03)	77	75 (03)
VE-204	59	55 (03)	73	73 (01)	75	74 (02)
VE-205	58	57 (02)	72	70 (01)	77	76 (02)
VE-206	61	63 (02)	74	75 (01)	78	77 (01)
VE-207	61	64 (03)	74	72 (02)	76	77 (02)
VE-208	59	56 (03)	74	71 (02)	75	75 (01)
VE-209	61	60 (01)	74	71 (03)	76	74 (03)
VE-210	61	60 (02)	74	74 (01)	79	79 (01)
VE-211	59	58 (03)	73	73 (01)	76	74 (03)
VE-212	69	65 (02)	79	79 (02)	80	81 (02)
VE-213	59	56 (03)	74	74 (01)	75	75 (02)
VE-214	49	50 (01)	73	72 (02)	78	77 (01)
VE-215	61	59 (03)	73	72 (01)	75	75 (01)
VE-216	58	58 (01)	73	74 (01)	78	78 (01)
VE-217	58	57 (01)	73	72 (02)	79	80 (02)
VE-218	56	57 (02)	73	74 (02)	73	70 (03)
VE-219	58	56 (03)	73	73 (01)	77	78 (02)
VE-220	48	49 (01)	70	71 (02)	75	74 (02)
VE-221	59	59 (01)	73	70 (03)	75	74 (02)

^a^Numbers in parentheses are standard errors

**Fig. 3:**
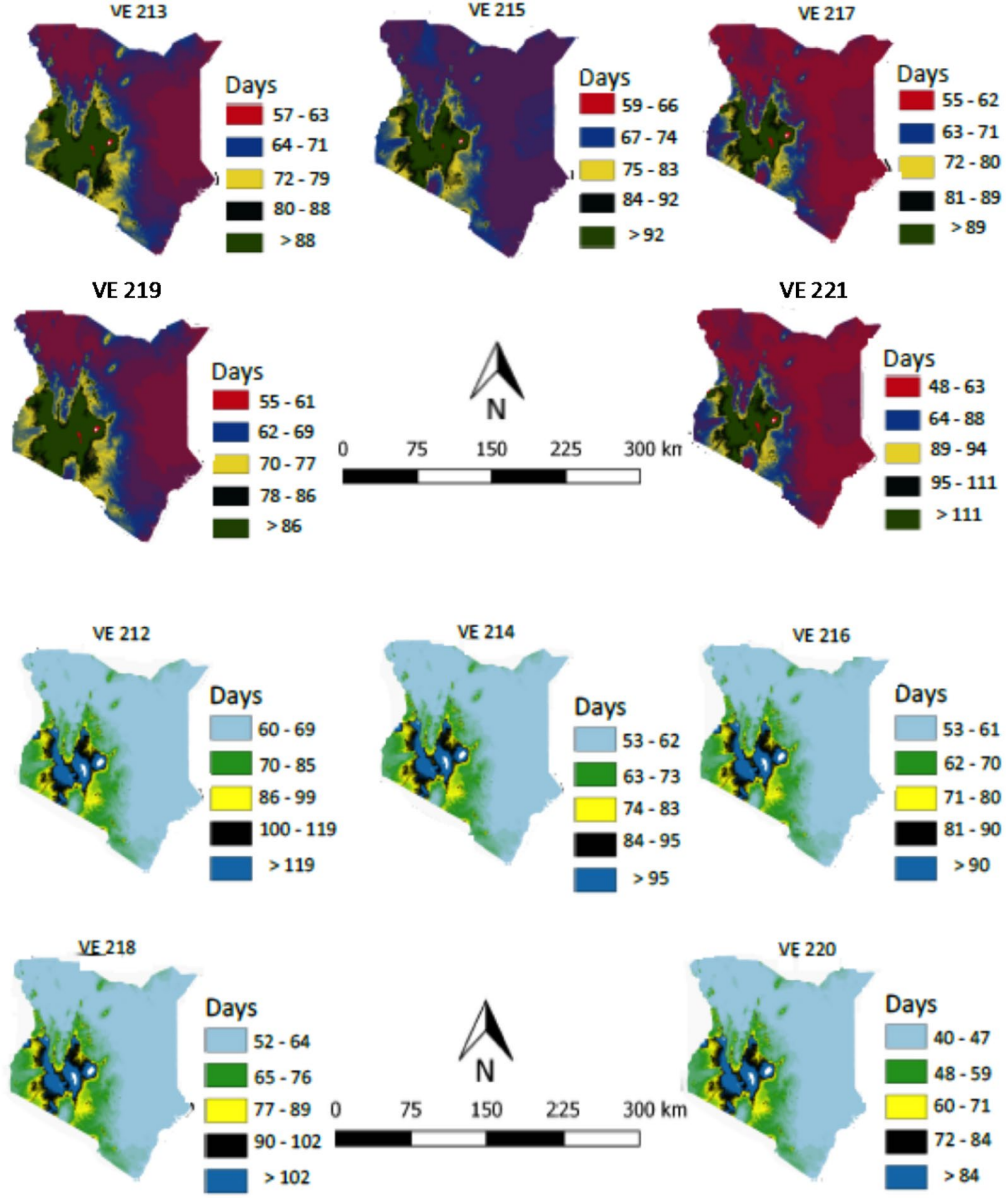
Maps of Kenya showing the number of days 10 maize varieties (VE 212, VE 213, VE 214, VE 215, VE 216, 217, VE 218, VE 219, VE 220 and VE 221) would take during each phase (vegetative or reproductive) of development in different agro-ecologies. The small white dots are areas with missing values of temperature

**Fig. 4:**
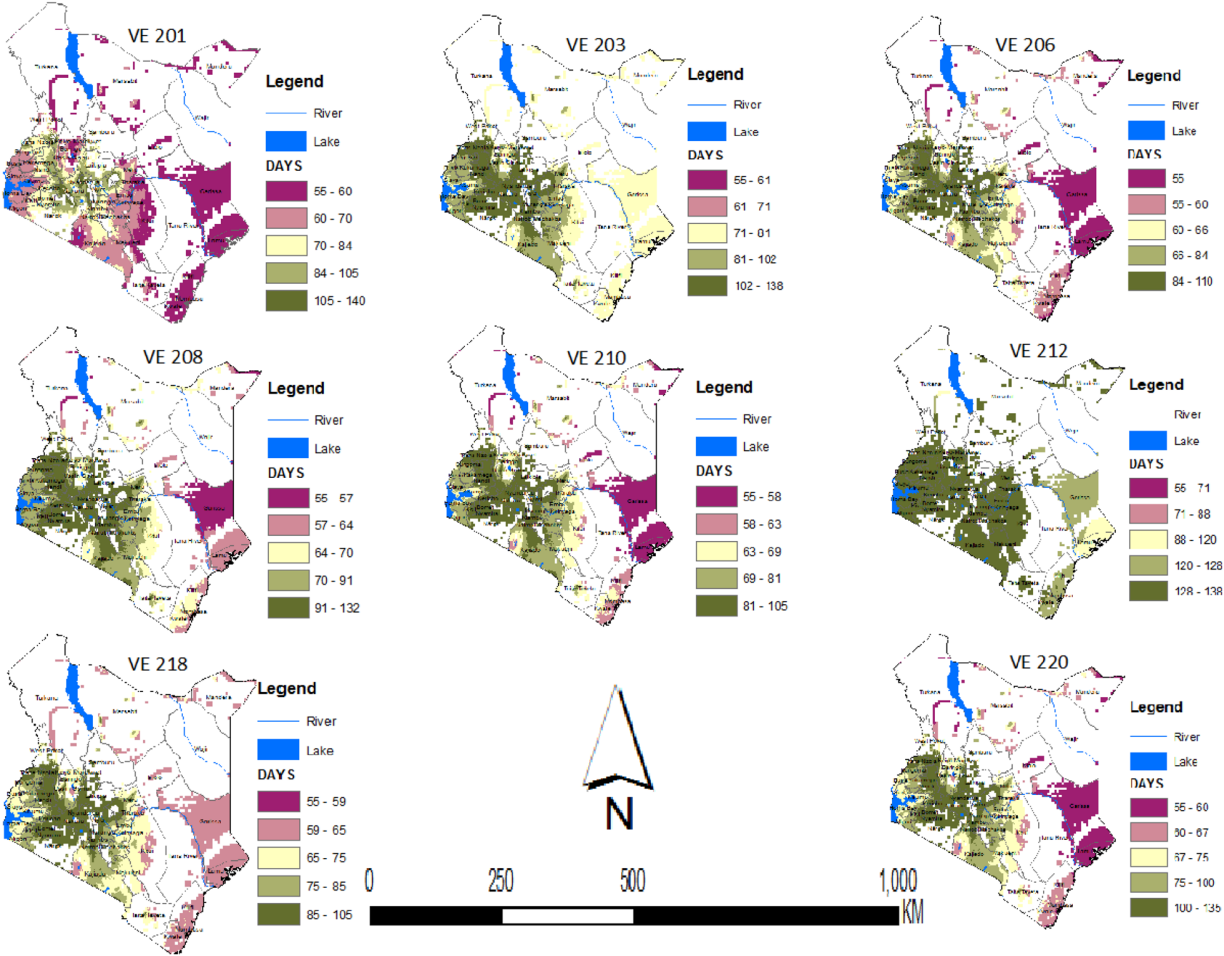
Maps of Kenya showing the number of days 10 maize varieties (VE 201, VE 203, VE 206, VE 208, VE 210, VE 212, VE 218, and VE 220) would take during each phase (vegetative or reproductive) of development in different agro-ecologies. This figure was obtained by masking the distribution map of suitable soil and weather conditions for growing maize with the phenology maps presented in Fig. 3 and in this figure

**Fig. 5:**
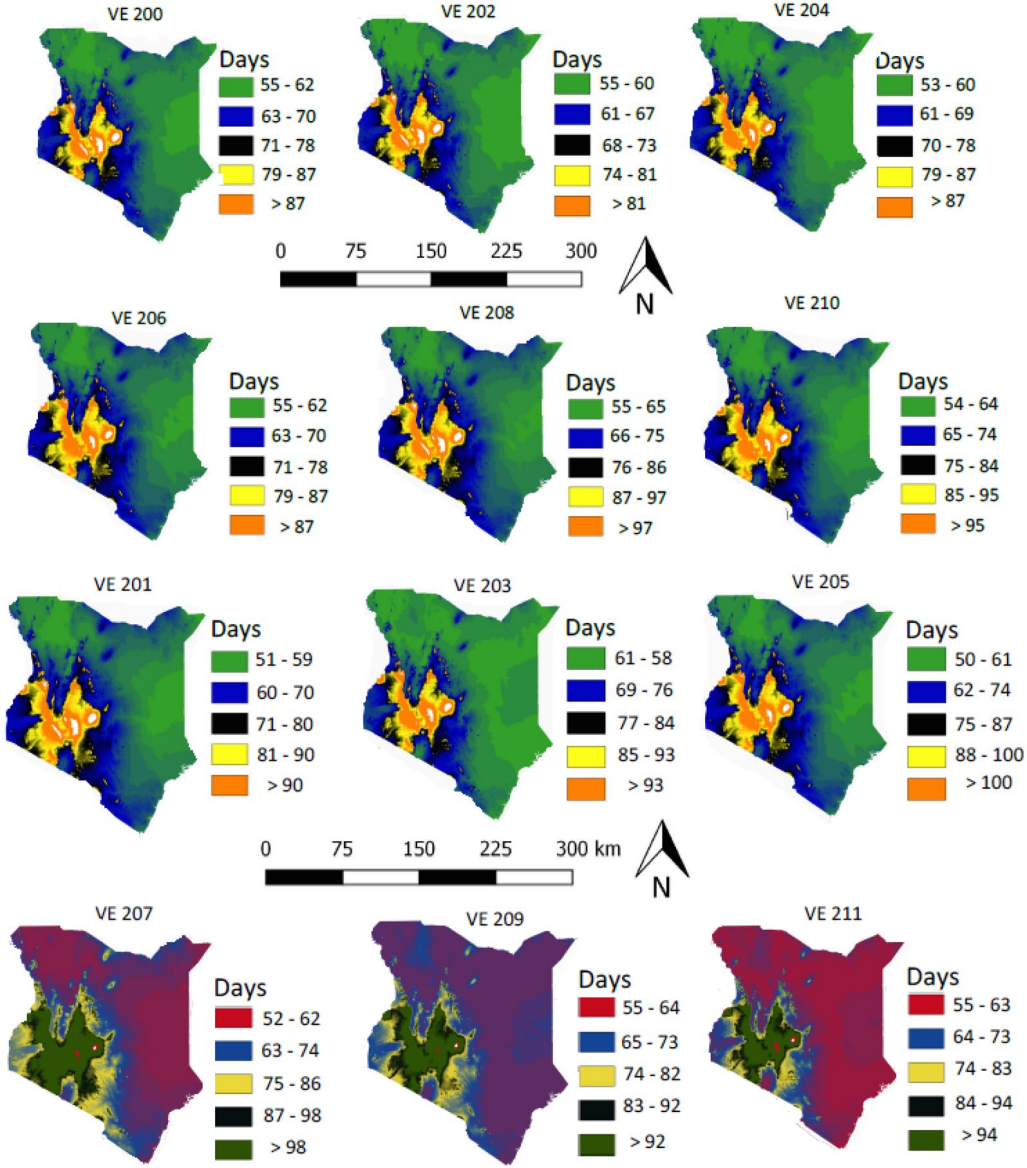
Maps of Kenya showing the number of days 12 maize varieties (VE 201, VE 202, VE 203, VE 204, VE 205, VE 206, VE 207, VE 208, VE 209, VE 210 and VE 211) would take during each phase (vegetative or reproductive) of development in different agro-ecologies. The small white dots are areas with missing values of temperature

As expected, maize development is predicted to take longer in the cooler mid-altitudes of Western Kenya and to be shorter in the warmer lowlands of the coastal areas. Out of the 22 varieties of maize analyzed here, the longest growing period from sowing to flowering was for VE 212, where most predicted values were > 105 days. The earliest variety was VE 220 at < 90 days in the majority of regions in Kenya. These maps show that all selected varieties of maize can be grown in most parts of Kenya, with a range of adaptation among varieties available (Figs. 3, 4). The phenology models for each variety were mapped spatially and then filtered or masked to represent areas where soil and weather conditions are suitable for growing maize (Fig. 5 for selected varieties).

## Discussion

This study had two major objectives, namely to develop a model framework to rigorously predict maize phenology from routine yield trials and to map adaptation spatially. Maize breeders conduct many yield trials each year in a wide range of locations representing target environments as well as ‘hot-spots’ for biotic or abiotic stresses [16]. In most, if not all, trials phenology, a key trait, is recorded. These standard data sets are available for predictive modeling and, as we have shown, are resources that can be successfully used to add value to the breeding process.

The maize plant requires a certain amount of heat units or thermal energy to transition between different phases of development [13, 33]. Because of yearly and within season variation in weather patterns, measuring the heat units accumulated over time provides a physiological time scale that is biologically more accurate than calendar days for predicting stages of development [13, 33]. Phenology models, characterized by a rate of development, are often applied to predict the timing of events in plant development. However, it is difficult to directly measure the rate of plant development [4]. Usually a relationship is established between the development rate (development rate is calculated as the inverse of development time), and the development time as the latter (e.g. flowering date) can be easily and routinely measured from field studies. The most critical step in the current study was the choice of a model among numerous thermal mathematical models that exist to describe the developmental response of plants and insects to temperature. No standard method exists to choose between competing models and the modeling framework developed here provides a solution. The decision on choice of a model should not be based on statistics alone but a combination of statistics and scientist’s experience and skills to identify an appropriate model that provides the best biologically meaningful and statistically significant parameters. The model of Briere et al. [23] described well the development rate of the selected maize varieties. This finding was interesting because, to the best of our knowledge, the Briere model has not been used in the context of plant development. The model has always been applied to describe the temperature dependent development rate of insect life stages [31]. The model provided estimates of number of days from emergence to the end of the vegetative phase that closely matched the recorded data at the three locations. This suggested that this model is suitable for modeling growth of tropical maize. The model has several features: it requires a small number of parameters making it easy to fit and the formulation allows the global minimum of the loss function to be rapidly reached. The parameters *T*_*b*_ and *T*_*m*_ have biological definitions that provide smooth curves with adequate approximation of biological processes. The curve captured maize response to temperature within a large range of temperature values. The selected model includes numerous features such as the dependency at low temperatures, the positive linear dependence at intermediate temperatures, the parabola response across the optimum temperatures and the negative linear dependency at high temperatures [10, 34]. Many of these features are not found among the eight temperature dependent models used for predicting the phenology of maize as described in [11]. This study showed that the accuracy of the models applied was mainly associated with the temperature response across a selected range rather than *T*_*b*_ and *T*_*m*_. Herein we argue that such association may not always be adequate as the physiological process of maize crop development is illustrated by gradual variation of organs and tissues stemming in gradients [19]. During maize development, the temperature responses are absolute and not relative; therefore shifting temperature beyond a set limit can stimulate an immediate effect, which may not be evident if the value of the temperature was unchanged or remained below or above the threshold. In other words the threshold value of temperature is an important variable for accurately predicting maize phenology and should not be omitted but carefully estimated through the smoothness of the model we have proposed [19].

Although numerous efforts have been made by researchers to suggest mathematical expressions to describe the relationship between the timing of events during maize development and environmental temperature [15, 18], few attempts were made on the mapping of phenological development in SSA. Simulation of the phenological development of wheat and maize at global scale was conducted by [15]. The authors applied the concept of heat units, which assumes the accumulation of daily temperature above *T*_*b*_. Because the model was primarily developed for the temperate regions, the equation included an incremental modification of the heat units due to the effect of photoperiod. Using crop calendars and large-scale pattern of phenological characteristics of varieties, a spatial prediction of the length of growing season for each crop was produced. The results revealed an over and under estimation by 0.5–1.5 months of the duration of cropping period of maize [15]. Such outcome may be explained by the fact that a linear model (degree-day), which lacks the ability to capture the nonlinearity in the processes that govern plant development, was used. In addition, the model was projected globally; ignoring that in tropical regions the effects of the sensitivity to day length (photoperiod) is negligible on maize phenology. We suggest that the calibration of model should be conducted for a defined area of interest, as a single model is unlikely to work everywhere.

Taking into account that maize crop is grown across large agro-ecologies in Kenya, and elsewhere in Africa, it was important in this study to adopt a framework that could provide some level of knowledge at agro-ecological and national level. The mapping framework used readily available open-access temperature (and other variables if desired) data and could be used for other crops and research questions. Therefore, to scale up our results, a bottom-up approach in which a number of sites were selected for the analysis of the model followed by mapping was adopted. During the mapping step the temperature applied contained wider ranges than those used at individual trial locations, which helped to estimate the length of growing period for each variety of maize in the whole of Kenya. Such outcome is of high importance to farmers as it provides location specific and accurate duration of events during the growing of maize, which could guide in management planning. The predictive mapping framework could be used at a range of scales depending on the user and their needs. For example, extension workers may want to know what variety performs best at a particular location. Input dealers, on the other hand, may want to know what range of varieties to stock for their market area.

### Implications of the study and conclusions

Phenology models are important analytical tools for predicting, evaluating, and understanding the length of crop growing duration under diverse environmental conditions. Linking the model with climate drivers such as temperature, and scaling out through predictive mapping, permitted us to estimate the phase duration of specific maize varieties throughout areas where maize is and can be grown in Kenya. Furthermore, these predictive models can be developed for other species and also used with future climate scenarios, to assess climate change impacts [35] and future breeding targets.

In East Africa, information on maize seed packages is usually very limited; with varieties being categorized in imprecise altitude classes (low, medium, high) and/or maturity groups (early, medium, late), neither of which are predictive. This study provided predictive, and hence location specific length of growing periods for 22 maize varieties. This information can help farmers in two ways. First, information on when varieties will mature can help farmers select the best variety for their location and growing season. Second, phonological predictions can be used to predict the timing of key stages of development that can be used to provide advice on the timing key events such weeding and application of fertilizer that will lead to improved yields. In future we intend to compact the maps and include additional features for each variety of maize (name, type, color, potential yield, maturity class, ecology, resistance to diseases/pests, tolerance to abiotic stresses, year and company of release) to be provided by breeders and specific country variety release institutions to develop a tool and smart phone applications that we hope will contribute to increased maize production in sub-Saharan Africa.

## Additional file

**Additional file 1: Table S1.** Summary of the mathematical expressions of the 82 models tested in the study.
