# Supplementary material for: Methodological approach for predicting and mapping the phenological adaptation of tropical maize (Zea mays L.) using multi-environment trials
Source: Plant Methods. 2018 Dec 7;14:108. doi: 10.1186/s13007-018-0375-7 (PMC6284281; doi:10.1186/s13007-018-0375-7)
Supplement: Supplementary file 1 — Additional file 1: Table S1. Summary of the mathematical expressions of the 82 models tested in the study. [file 13007_2018_375_MOESM1_ESM.docx]

**Supplemental Table 1:** Summary of the mathematical expressions of the 82 models tested

| **ID** | **Name** | **Equation** | **Comment** | **Reference** |
| --- | --- | --- | --- | --- |
| 1 | Sharpe & DeMichele 1 | $r\left(T\right)=\frac{p'.\frac{T}{T_{0}'}.e^{\left[\frac{\Delta H_{A}}{R}\left(\frac{1}{T_{0}'}-\frac{1}{T}\right)\right]}}{1+e^{\left[\frac{\Delta H_{L}}{R}\left(\frac{1}{T_{L}}-\frac{1}{T}\right)\right]}+e^{\left[\frac{\Delta H_{H}}{R}\left(\frac{1}{T_{H}}-\frac{1}{T}\right)\right]}}$ $p'=a+b*T_{o}'$ $T_{o}'=\frac{\Delta H_{L}-\Delta H_{H}}{R*\log\left(-\frac{\Delta H_{L}}{\Delta H_{H}}\right)+\left(\frac{\Delta H_{L}}{T_{L}}\right)-\left(\frac{\Delta H_{H}}{T_{L}}\right)}$     | 5P |  |
| 2 | Sharpe & DeMichele 2 | $r\left(T\right)=\frac{p'.\frac{T}{T_{0}}.e^{\left[\frac{\Delta H_{A}}{R}\left(\frac{1}{T_{0}}-\frac{1}{T}\right)\right]}}{1+e^{\left[\frac{\Delta H_{L}}{R}\left(\frac{1}{T_{L}}-\frac{1}{T}\right)\right]}+e^{\left[\frac{\Delta H_{H}}{R}\left(\frac{1}{T_{H}}-\frac{1}{T}\right)\right]}}$ $p'=a+b*T_{o}$   | 6P |  |
| 3 | Sharpe & DeMichele 3 | $r\left(T\right)=\frac{p.\frac{T}{T_{0}}.e^{\left[\frac{\Delta H_{A}}{R}\left(\frac{1}{T_{0}}-\frac{1}{T}\right)\right]}}{1+e^{\left[\frac{\Delta H_{L}}{R}\left(\frac{1}{T_{L}}-\frac{1}{T}\right)\right]}+e^{\left[\frac{\Delta H_{H}}{R}\left(\frac{1}{T_{H}}-\frac{1}{T}\right)\right]}}$ | 7P | Sharpe and DeMichele  1977 |
| 4 | Sharpe & DeMichele 4 | $r\left(T\right)=p.\frac{T}{T_{0}}.e^{\left[\frac{\Delta H_{A}}{R}\left(\frac{1}{T_{0}}-\frac{1}{T}\right)\right]}$ | 3P |  |
| 5 | Sharpe & DeMichele 5 | $r\left(T\right)=\frac{p.\frac{T}{T_{0}}.e^{\left[\frac{\Delta H_{A}}{R}\left(\frac{1}{T_{0}}-\frac{1}{T}\right)\right]}}{1+e^{\left[\frac{\Delta H_{L}}{R}\left(\frac{1}{T_{L}}-\frac{1}{T}\right)\right]}}$ | 5P |  |
| 6 | Sharpe & DeMichele 6 | $r\left(T\right)=\frac{p.\frac{T}{T_{0}}.e^{\left[\frac{\Delta H_{A}}{R}\left(\frac{1}{T_{0}}-\frac{1}{T}\right)\right]}}{1+e^{\left[\frac{\Delta H_{H}}{R}\left(\frac{1}{T_{H}}-\frac{1}{T}\right)\right]}}$ | 5P |  |
| 7 | Sharpe & DeMichele 7 | $r\left(T\right)=p'.\frac{T}{T_{0}}.e^{\left[\frac{\Delta H_{A}}{R}\left(\frac{1}{T_{0}}-\frac{1}{T}\right)\right]}$ $p'=a+b*T_{o}'$   | 2P |  |

| 8 | Sharpe & DeMichele 8 | $r\left(T\right)=\frac{p'.\frac{T}{T_{0}}.e^{\left[\frac{\Delta H_{A}}{R}\left(\frac{1}{T_{0}}-\frac{1}{T}\right)\right]}}{1+e^{\left[\frac{\Delta H_{L}}{R}\left(\frac{1}{T_{L}}-\frac{1}{T}\right)\right]}}$ $p'=a+b*T_{o}'$   | 4P |  |
| --- | --- | --- | --- | --- |
| 9 | Sharpe & DeçMichele 9 | $r\left(T\right)=\frac{p'.\frac{T}{T_{0}}.e^{\left[\frac{\Delta H_{A}}{R}\left(\frac{1}{T_{0}}-\frac{1}{T}\right)\right]}}{1+e^{\left[\frac{\Delta H_{H}}{R}\left(\frac{1}{T_{H}}-\frac{1}{T}\right)\right]}}$ $p'=a+b*T_{o}'$   | 4P |  |
| 10 | Sharpe & DeMichele 10 | $r\left(T\right)=\frac{p.\frac{T}{T_{0}'}.e^{\left[\frac{\Delta H_{A}}{R}\left(\frac{1}{T_{0}}-\frac{1}{T}\right)\right]}}{1+e^{\left[\frac{\Delta H_{L}}{R}\left(\frac{1}{T_{L}}-\frac{1}{T}\right)\right]}+e^{\left[\frac{\Delta H_{H}'}{R}\left(\frac{1}{T_{H}'}-\frac{1}{T}\right)\right]}}$ $H_{H}'=1$ $T_{H}'=1$    | 4P | Sporleder *et al.* (2004) |
| 11 | Sharpe & DeMichele 11 |  | 6P |  |
| 12 | Sharpe & DeMichele 12 | $P'=a+b*T_{0}$   | 5P |  |
| 13 | Sharpe & DeMichele 13 | $r(T)=\frac{P*\frac{T}{T_{0}}*e^{\left[\frac{\Delta H_{A}}{R}\left(\frac{1}{T_{0}}-\frac{1}{T}\right)\right]}}{1+e^{\left[\frac{\Delta H_{L}}{R}\left(\frac{1}{T_{L}}-\frac{1}{T}\right)\right]}}$ | 4P |  |
| 14 | Sharpe & DeMichele 14 | $r(T)=\frac{P*\frac{T}{T_{0}}*e^{\left[\frac{\Delta H_{A}}{R}\left(\frac{1}{T_{0}}-\frac{1}{T}\right)\right]}}{1+e^{\left[\frac{\Delta H_{H}}{R}\left(\frac{1}{T_{H}}-\frac{1}{T}\right)\right]}}$ | 4P |  |

| 15 | Deva 1 | $r(T)=b\left(T-T_{\min}\right)$ T ≥ Tmax $r(T)=0$  T < Tmax | 2P | Dallwits and Higgins  1992 |
| --- | --- | --- | --- | --- |
| 16 | Deva 2 | $r(T) = b_{1} \left(10^{-v^{2}}\right)\left(1-b_{5}+b_{5}v^{2}\right)$ $v = \frac{\left(u+e^{b_{4}\left(u\right)}\right)}{c_{2}}$ $u = \frac{\left(T-b_{3}\right)}{\left(b_{3}-b_{2}\right)}-c_{1}$ $c_{1} = \frac{1}{1+0.28b_{4}+0.72\ln\left(1+b_{4}\right)}$ $c_{2} = \frac{1+b_{4}}{1+1.5b_{4}+0.39b_{4}^{2}}$      | 5P | Dallwits and Higgins  1992 |
| 17 | Logan 1 | $r(T)=Y*(e^{p*T}-e^{\left(p*T_{\max}-\frac{\left(T_{\max}-T\right)}{v}\right)})$ | 4P | Longan 1976 |
| 18 | Logan 2 | $r(T)=\alpha\left(\frac{1}{1+k*e^{-p*T}}-e^{-\frac{\left(T_{\max}-T\right)}{v}}\right)$ | 5P | Longan 1976 |
| 19 | Briere 1 | $r(T)=a*T\left(T-T_{o}\right)\left(\sqrt{T_{\max}-T}\right)$ | 3P  T ≤ Tmax | Briere et al.  1999 |
| 20 | Briere 2 | $r(T)=a*T\left(T-T_{o}\right)\left(T_{\max}-T\right)^{d}$ | d Є Z**→**T Є R  d∉ Z**→**T≤Tmax | Briere et al.  1999 |
| 21 | Stinner 1 | $r(T)=\frac{R_{\max}\left(1+e^{k_{1}+k_{2}\left(T_{opc}\right)}\right)}{1+e^{k_{1}+k_{2}\left(T\right)}}$ | 4P | Stinner et al.  1974 |
| 22 | Hilber & logan 1 | $r(T)=Y\left(\frac{T^{2}}{T^{2}+d^{2}}-e^{-\frac{\left(T_{\max}-T\right)}{v}}\right)$ | 4P | Hilber and logan 1983 |
| 23 | Lactin 1 | $r(T)=e^{p*T}-e^{-\frac{\left(p*T_{l}-\left(T-T_{l}\right)\right)}{dt}}+\lambda$ | 4P | Lactin et al.  1995 |
| 24 | Linear | $r(T)=a+b*T$ | 2P |  |
| 25 | Exponential simple | $r(T)=b_{1}*e^{b_{2}*T}$ | 2P |  |
| 26 | Tb model | $r(T)=sy*e^{\left(b\left(T-T_{b}\right)-e^{\left(\frac{b\left(T-T_{b}\right)}{DT_{b}}\right)}\right)}$ | 4P |  |
| 27 | Exponential model | $r(T)=sy*e^{b\left(T-T_{b}\right)}$ | 3P |  |
| 28 | Exponential | $r(T)=e^{b\left(T-T_{\min}\right)}-1$ | 2P |  |
| 29 | Ratkowsky 1 | $r(T)=b\left(T-T_{b}\right)^{2}$ | 2P | Ratkowsky et al. 1982 |
| 30 | Davidson | $r(T)=\frac{k}{1+e^{a-bT}}$ | 2P | Davidson  1942, 1944 |

| 31 | Pradham | $r(T)=R_{m}e^{\left[\frac{-1}{2}\left(\frac{T-T_{m}}{T_{\sigma}}\right)^{2}\right]}$ | 3P | Pradham 1945 |
| --- | --- | --- | --- | --- |
| 32 | Angilletta Jr. | $r(T)=a*e^{\left[-\frac{1}{2}\left(\frac{\left\vert T-b\right\vert}{c}\right)^{d}\right]}$ | 4P | Angilletta Jr. 2006 |
| 33 | Stinner 2 | $r(T)=\frac{MaxDevRate*e^{k_{1}+k_{2}T_{opt}}}{1+e^{k_{1}+k_{2}T}}$ | 4P | Stinner  1974 |
| 34 | Hilbert | $r(T)=\Psi\left[\frac{(T-T_{b})^{2}}{((T-T_{b})+D^{2})}\right]-e^{-(\frac{T_{m}-(T-T_{b})}{\Delta T})}$ | 5P | Hilbert and Logan 1983 |
| 35 | Lactin 2 | $r(T)=e^{\rho T}-e^{(\rho T_{Max}-\frac{T_{Max}-T}{\Delta})}$ | - 5P | - Lactin et al. 1995 |
| 36 | Anlytis 1 | $r(T)=P\delta^{n}(1-\delta)^{m}$ $\delta=\frac{T-T_{mim}}{T_{\max}-T_{\min}}$  | 5P  n Є Z**→**T Є R  mЄ Z**→**T Є R  n∉ Z**→**T≥Tmin  m∉Z**→**T≤Tmx | Analysis 1977 |
| 37 | Anlytis 2 | $r(T)=[P\delta^{n}(1-\delta)]^{m}$ $\delta=\frac{T-T_{mim}}{T_{\max}-T_{\min}}$  | 5P  n Є Z**→**T Є R  mЄ Z**→**T Є R  n∉ Z**→**T≥Tmin  m∉Z**→**T≤Tmx | Analysis 1980 |
| 38 | Anlytis 3 | $r(T)=a(T-T_{\min})^{n}(T_{\max}-T)^{m}$ | 5P  n Є Z**→**T Є R  mЄ Z**→**T Є R  n∉ Z**→**T≥Tmin  m∉Z**→**T≤Tmx | Analysis 1977 |
| 39 | Allahyari | $r(T)=P\delta^{n}(1-\delta^{m})$ $\delta=\frac{T-T_{mim}}{T_{\max}-T_{\min}}$  | 5P  n Є Z**→**T Є R  mЄ Z**→**T Є R  n∉ Z**→**T≥Tmin  m∉Z**→**T≥Tmin | Allahyari 2005 |
| 40 | Briere 3 | $r(T)=a(T-T_{b})(T_{L}-T)^{\frac{1}{2}}$ | 3P | Briere et al.  1999 |
| 41 | Briere 4 | $r(T)=a(T-T_{b})(T_{L}-T)^{\frac{1}{n}}$ T ≤ T_L_ | 3P | Briere et al.  1999 |

| 42 | Kontodimas 1 | $r(T)=a(T-T_{\min})^{2}(T_{\max}-T)$ | 3P | Kontodimas 2004 |
| --- | --- | --- | --- | --- |
| 43 | Kontodimas 2 | $r(T)=\frac{2}{D_{\min}(e^{K(T-T_{opt})}+e^{-\lambda(T-T_{opt})})}$ | 4 P | Kontodimas 2004 |
| 44 | Kontodimas 3 | $r(T)=\frac{T*e^{\left(a-\frac{b}{T}\right)}}{1+e^{\left(c-\frac{d}{T}\right)}+e^{\left(f-\frac{g}{T}\right)}}$ | 6P | Kontodimas 2004 |
| 45 | Ratkowsky 2 | $r(T)=\left[a\left(T-T_{\min}\right)\left(1-e^{b(T-T_{\max})}\right)\right]^{2}$ | 4P | Ratkowsky et al. 1982 |
| 46 | Janish 1 | $r(T)=\frac{2}{D_{\min}(e^{K(T-T_{opt})}+e^{-K(T-T_{opt})})}$ | 3P |  |
| 47 | Janish 2 | $r(T)=\frac{2C}{a^{(T-Tm)}+b^{(T_{m}-T)}}$ | 3P |  |
| 48 | Tanigoshi | $r(T)=a_{0}+a_{1}T+a_{2}T^{2}+a_{3}T^{3}$ | 4P | Tanigoshi and Browne 2004 |
| 49 | Wang-Lan-Ding | $r(T)=k\frac{\left(1-e^{-a(T-T_{\min})}\right)\left(1-e^{b(T-T_{\max})}\right)}{1+e^{-r(T-c)}}$ | 7P | Wang et al. 1982 |
| 50 | Stinner 3 | $r(T)=\frac{c_{1}}{1+e^{\left(k_{1}+k_{2}*T\right)}}$ | 3P |  |
| 51 | Stinner 4 | $r(T)=\frac{c_{1}}{1+e^{\left(k_{1}+k_{2}*T\right)}}+\frac{c_{2}}{1+e^{\left(k_{1}+k_{2}\left(2*To-T\right)\right)}}$ | 5P |  |
| 52 | Logan 3 | $r(T)=sy*e^{\left(b\left(T-T_{\min}\right)-e^{\left(b*T_{\max}-\frac{\left(T_{\max}-\left(T-T_{\min}\right)\right)}{Dtb}\right)}\right)}$ | 5P |  |
| 53 | Logan 4 | $r(T)=\alpha\left(\frac{1}{1+k*e^{-b\left(T-T_{\min}\right)}}-e^{-\frac{\left(T_{\max}-\left(T-T_{\min}\right)\right)}{Dt}}\right)$ | 5P |  |

| 54 | Logan-5 | $r(T)=\alpha\left(\frac{1}{1+k*e^{-b\left(T\right)}}-e^{-\frac{\left(T_{\max}-T\right)}{Dt}}\right)$ | 6P |  |
| --- | --- | --- | --- | --- |
| 55 | Hilber & logan 2 | $r(T)=\psi\left(\frac{T^{2}}{T^{2}+D}-e^{-\frac{\left(T_{\max}-T\right)}{Dt}}\right)$ | 4P |  |
| 56 | Hilber & logan 3 | $r(T)=\psi\left(\frac{\left(T-T_{\min}\right)^{2}}{\left(T-T_{\min}\right)^{2}+D}-e^{-\frac{\left(T_{\max}-\left(T-T_{\min}\right)\right)}{Dt}}\right)+\theta$ | 6P |  |
| 57 | Taylor | $r(T)=r_{m}*e^{-\frac{1}{2}\left(-\frac{\left(T-T_{opt}\right)}{T_{roh}}\right)^{2}}$ | 3P |  |
| 58 | Lactin 3 | $r(T)=e^{\rho*T}-e^{(\rho*T_{Max}-\frac{T_{Max}-T}{\Delta})}+\lambda$ | 4P |  |
| 59 | Sigmoid or Logistic | $r(T)=\frac{c_{1}}{1+e^{a+b*T}}$ | 3P |  |

| 60 | MAIZSIM | $r\left( T \right)=\mathrm{Rmax}\left( \frac{Tceil-T}{Tceil-Topc} \right)\left( \left( \frac{T}{\mathrm{Topc}} \right)^{\frac{\mathrm{Topc}}{Tceil-Topc}} \right)$ | 3P | (Kim et al., 2012) |
| --- | --- | --- | --- | --- |
| 61 | Enzymatic Response | $r\left( T \right)=\frac{51559240052.T.{exp}^{\left( \frac{-73900}{8.314.T} \right)}}{1+\left( {exp}^{\left( \frac{-73900}{8.314T} \right)} \right)^{\left( \frac{alpha*(1-T)}{To} \right)}}$ | 2P |  |
| 62 | beta function | $r\left( T \right)= {exp}^{\left( k \right)}.\left( \left( T-Tb \right)^{alpha} \right).\left( \left( Tc-T \right)^{betas} \right)$ | 5P |  |
| 63 | Wang et Engel | $r\left( T \right)=\frac{2\left( \left( T-Tmin \right)^{\left( \frac{\log\left( 2 \right)}{\log\left( \frac{Tmax-Tmin}{Topt-Tmin} \right)} \right)} \right)\left( \left( Topt-Tmin \right)^{\left( \frac{\log\left( 2 \right)}{\log\left( \frac{Tmax-Tmin}{Topt-Tmin} \right)} \right)} \right)-\left( \left( T-Tmin \right)^{\left( \frac{2*\log\left( 2 \right)}{\log\left( \frac{Tmax-Tmin}{Topt-Tmin} \right)} \right)} \right)}{\left( \left( Topt-Tmin \right)^{\left( \frac{2*\log\left( 2 \right)}{\log\left( \frac{Tmax-Tmin}{Topt-Tmin} \right)} \right)} \right)}$ | 3P | (Wang et Engel, 1998) |
| 64 | Richards | $r\left( T \right)=\frac{\mathrm{Yasym}}{\left( 1+v.{exp}^{\left( -k.(T-Tm) \right)} \right)^{1/v}}$ | 4P | Richards (1959) |
| 65 | Gompertz | $r\left( T \right)=Yasym*{exp}^{\left( -{exp}^{\left( -k*(x-Tm) \right)} \right)}$ | 3P | Gompertz (1825) |
| 66 | Beta 1 | $r\left( T \right)=\mathrm{Rmax}* \left( 1+\frac{Tmax-x}{Tmax-Topt} \right). \left( \frac{T}{Tmax} \right)^{\left( \frac{Tmax}{Tmax-Topt} \right)}$ | 3P | (Yin et al.,2003a) |
| 67 | Q10 function | $r\left( T \right)={Q10}^{\left( \frac{T-Tref}{10} \right)}$ | 2P |  |
| 68 | Ratkowsky 3 | $r\left( T \right)=\frac{\left( T-Tmin \right)^{2}}{\left( Tref-Tmin \right)^{2}}$ | 2p | Ratkowsky et al.(1982) |
| 69 | Beta 2 | $r\left( T \right)=Rmax. \left( \frac{Tmax-T}{Tmax-Topt} \right)\left( \frac{T}{Tmax} \right)^{\left( \frac{Tmax}{Tmax-Topt} \right)}$ | 3P | (Yin et al. 1995) |
| 70 | Bell curve | $r\left( T \right)=Yasym. {exp}^{\left( a\left( T-Topt \right)^{2}+b\left( T-Topt \right)^{3} \right)}$ | 4P |  |
| 71 | Gaussian function | $r\left( T \right)=Yasym. {exp}^{\left( -0.5.\left( \frac{T-Topt}{b} \right)^{2} \right)}$ | 3P |  |
| 72 | Beta 3 | $r\left( T \right)=\mathrm{Rmax}.\left( \left( \frac{Tmax-T}{Tmax-Topt} \right)* \left( \left( \frac{T-\mathrm{Tmin}}{Topt-Tmin} \right)^{\left( \frac{Topt-Tmin}{Tmax-Topt} \right)} \right) \right)$ | 4P | (Yan et Hunt, 1999) |
| 73 | Expo first order plus logistic | $r\left( T \right)=Yo.\left( 1- {exp}^{\left( kT \right)} \right)+bT$ | 3P | Gilles and price 2011) |
| 74 | Beta 4 | $r\left( T \right)=Yb+ \left( Rmax-Yb \right).\left( 1+ \frac{Tmax-T}{Tmax-Topt} \right)* \left( \left( \frac{T-\mathrm{Tmin}}{Tmax-Tmin} \right)^{\left( \frac{Tmax-Tmin}{Tmax-Topt} \right)} \right)$ | 5P | (Yin et al., 2003) |
| 75 | Beta 5 | $r\left( T \right)=\mathrm{Rmax}\frac{\left( 2.Tmax-T \right). T}{\mathrm{Tmax}^{2}}$ | 2P | (Yin et al., 2003) |
| 76 | Beta 6 | $r\left( T \right)=\mathrm{Rmax}\frac{\left( 3.Tmax-2.T \right).T^{2}}{\mathrm{Tmax}^{3}}$ | 2P | (Yin et al., 2003) |
| 77 | Beta 7 | $r\left( T \right)=\mathrm{Rmax}.\left( 1-\left( 1+\left( \frac{Tmax-T}{Tmax-Topt} \right) \right)* \left( \left( \frac{T}{Tmax} \right)^{\left( \frac{Tmax}{Tmax-Topt} \right)} \right) \right)$ | 3P | (Yin et al., 2009) |
| 78 | Modified exponential | $r\left( T \right)=exp\left( a+bT.(1-0.5T/Topt) \right)$ | 3P | (O'Connell 1990) |
| 79 | Lorentzian 3-parameter | $r\left( T \right)=\frac{a}{\left( 1+\left( \frac{T-Topt}{b} \right)^{2} \right)}$ | 3P |  |
| 80 | Lorentzian 4-parameter | $r\left( T \right)=\frac{Yopt+a}{\left( 1+\left( \frac{T-Topt}{b} \right)^{2} \right)}$ | 4P |  |
| 81 | Log normal 3-parameter | $r\left( T \right)=a.{exp}^{\left( -0.5*\left( \frac{\log\left( \frac{T}{\mathrm{Topt}} \right)}{b} \right)^{2} \right)}$ | 3P |  |
| 82 | Pseudo-voigt 4 parameter | $r\left( T \right)=a.\left( \left( \frac{k}{1+\left( \frac{T-Topt}{b} \right)^{2}} \right)+\left( 1-k \right).{exp}^{\left( -0.5*\left( \frac{\left( T-\mathrm{Topt} \right)}{b} \right)^{2} \right)} \right)$ | 4P |  |

- *T* temperature in Degree Celcius

*- r(T)* development rate at temperature *T*

R= 1987 cal degree (-1) mol(-1)
